# Editorial: Root structure and function adapting to climate change

**DOI:** 10.3389/fpls.2024.1365263

**Published:** 2024-01-23

**Authors:** Yinglong Chen, Jairo A. Palta

**Affiliations:** School of Agriculture and Environment, and UWA Institute of Agriculture, The University of Western Australia, Perth, WA, Australia

**Keywords:** climate change, root structure, root functions, environmental stress, stress physiology, adaptation mechanisms

Global climate change has had a profound impact on ambient temperatures and on the amount and seasonal distribution of rainfall, leading to a reduction in soil moisture and an exacerbation of heatwaves (IPCC, 2007). Projections indicate that by the end of the century, temperatures are expected to rise by 2.6 to 4.8°C compared to current levels (The Royal Society and National Academy of Sciences, 2020). In addition to increases in temperature and reduction in precipitation, atmospheric CO_2_ concentrations are also on the rise. The increased frequency of extreme weather events, such as soil warming, droughts, flooding, heatwaves, and wildfires, is contributing to a challenging environment for crop production and ecosystem management (Pugnaire et al., 2019; Calleja-Cabrera et al., 2020; IPCC, 2007; Lynch and Brown, 2012; Chen et al., 2015; Hazman and Brown, 2018; Malhi et al., 2020 Muluneh, 2021). Effectively addressing these challenges requires the implementation of mitigation measures, including the identification of genetic diversity, fostering adaptive phenotypic plasticity, and developing new crop varieties (Anderson and Song, 2020; Malhi et al., 2020; Brooker et al., 2022). This approach is crucial for enhancing the resilience of crops and ecosystems in the face of ongoing climate changes.

The plant root system serves as the primary organ responsible for foraging and acquiring nutrients and water from the soil, making it the initial and most sensitive target of climate change (Lynch and Brown, 2012; Chen et al., 2015). However, both the form and function of a root system are susceptible to changes in the environment (Hazman and Brown, 2018; Brooker et al., 2022). Understanding how the form and function of a root system contribute to plant growth, development, and productivity under various environmental stresses is crucial, especially given the vulnerability of root systems to changing conditions and the intricate interplay of these factors.

The root growth response to soil water availability was assessed in the field in 200 soybean accessions over a span of three years (Bui et al.). The investigation evaluated genetic variation in root morphological traits during the early growth stage and identified key root traits, such as root dry weight, total root length and root volume, contributing to improve water use efficiency. Traits such as root average diameter, number of root tips, and secondary lateral root density responded early to irrigation conditions. The studies also identified certain genotypes that demonstrated both high stability and strong growth performance across different water treatments and years, suggesting a robust root system of soybean varieties under varying soil moisture conditions.

In a Polyethylene Glycol (PEG)-6000-induced water stress experiment, 19 genes were identified directly participating in abscisic acid (ABA) metabolism, suberization, and aquaporin activity (Kim and Sung). The study suggests that there is a sophisticated regulation of the drought tolerance mechanisms in rice roots, extending up to the permanent wilting point (−1.5 MPa). ABA metabolism, suberization, and aquaporin activity may function independently and/or concurrently as a survival strategy against drought. The deposition of abundant suberin lamellae, coupled with passive water absorption facilitated by activated aquaporins and aerenchyma development, suggests that rice roots play a role in enhancing water retention within cells. These findings provide insight into the intricate regulatory networks governing water-associated mechanisms in rice under conditions of limited water availability.

A split-root systems with vertically partitioned water and nutrient availability was used to grow perennial grass *Panicum virgatum* aiming to determine how root systems specialize in acquiring multiple resources (Glass et al.). The differential responses in root elongation, root surface area, and branching, demonstrated the relationship between root form and function, indicating that the main function of the primary root system is for water acquisition while the function of lateral branches is nutrient uptake. Despite similar root elongation rates and root mass accumulation, the results highlight the existence of differential root functioning within perennial grasses, suggesting a fundamental relationship observed in various plant functional types. These differential root responses to resource availability need to be incorporated into root growth models through parameters such as maximum root length and branching interval.

Developing nutrient-use efficient rice lines, particularly in the context of changing climate and depleting resources, is vital. The study of Padmashree et al. phenotyped 118 rice lines for seedling vigor, root-related traits, and yield under both irrigated and aerobic conditions. Based on SSR markers, genome-wide association studies (GWAS) were conducted to identify marker-trait associations (MTAs). Significant correlations were found between root traits, seedling vigor, and yield-related traits. The study identified consistent MTAs on chromosomes 2, 3, and 12, with functional genes related to transcription factors, auxin carriers, and amino acid transporters. The highlighted rice lines with desirable traits can be utilized in breeding programs to improve seedling vigor, yield-related traits under different conditions, and adaptability to water-saving technologies.

This Research Topic provides insights into the recent knowledge regarding the response and adaptation of root systems to the dynamic interplay of changing climates, in particular drought stress. It explored the intricate relationship between the form and function of root systems and their role in plant resilience, development, and productivity under the stresses imposed by environmental changes.

## Author contributions

YC: Conceptualization, Data curation, Formal Analysis, Funding acquisition, Investigation, Project administration, Resources, Supervision, Writing – original draft, Writing – review & editing. JP: Conceptualization, Formal Analysis, Writing – original draft, Writing – review & editing.
